# Surveillance of *de novo* head and neck cancer in liver transplant recipients allows an early diagnosis: a retrospective analysis of a surveillance program

**DOI:** 10.3389/fonc.2025.1465123

**Published:** 2025-03-13

**Authors:** Ivan Zambrano, Juan Alcalde, Mercedes Iñarrairaegui, Fernando Rotellar, J. Ignacio Herrero

**Affiliations:** ^1^ Department of Internal Medicine, School of Medicine, Universidad de Navarra, Pamplona, Spain; ^2^ Othorhinolaringology Department, Clínica Universidad de Navarra, Pamplona, Spain; ^3^ Liver Unit, Clínica Universidad de Navarra, Pamplona, Spain; ^4^ Centro de Investigación Biomédica en Red de Enfermedades Hepáticas y Digestivas (CIBERehd), Madrid, Spain; ^5^ Instituto de Investigación Sanitaria de Navarra (IdiSNA), Pamplona, Spain; ^6^ Department of Surgery, Clínica Universidad de Navarra, Pamplona, Spain

**Keywords:** liver tranpslant, immunosuppression, alcohol, head and neck (H&N) cancer, smoking - adverse effects

## Abstract

**Introduction:**

*De novo* head and neck cancer is a common and severe complication than can occur after liver transplantation. However, it is unclear whether surveillance can help detect and prevent this type of cancer in liver transplant recipients.

**Patients and methods:**

We retrospectively examined 119 transplanted patients who had a smoking history above 20 pack-years. These patients underwent yearly evaluations by an ear-nose-throat specialist.

**Results:**

Twelve of them (10.1%) were diagnosed with head and neck cancer. The most significant risk factor for developing head and neck cancer was having been transplanted for alcoholic liver disease. Of the 12 diagnosed cases, six cases were diagnosed at an early-intermediate stage (stages 0-II), five were at an advanced stage (including one patient who was diagnosed at his first surveillance visit and two who did not attend the surveillance visits), and tumor stage was unknown in one case. Three patients had cancer recurrences, all of them had continued smoking after their initial diagnosis. The five-year actuarial survival rate after the diagnosis of head and neck cancer was 65.6%.

**Discussion:**

Annual surveillance for head and neck cancer may allow for early diagnosis and better survival rates after cancer diagnosis.

## Introduction

*De novo* malignancies are a common complication and a leading cause of late mortality after liver transplantation (LT). LT recipients are at a higher risk of developing cancer, with a two-to-three-fold increased risk of malignancy compared to the general population of the same age and sex (1). The most commonly occurring post-transplant malignancies are post-transplant lymphoproliferative disease and non-melanoma skin cancer. Other types of solid malignancies arise less frequently.

Head and neck cancer (HNC) is one of the most frequent non-skin solid cancers diagnosed in LT recipients (2). Several studies have shown a higher incidence of HNC in LT recipients than in matched populations (3–5). Furthermore, overall survival after HNC diagnosis is worse in LT recipients than in the general population (6). Unfortunately, HNC is frequently diagnosed at an advanced stage in LT recipients, thus making their prognosis poor (6–8).

The International Liver Transplantation Society-Sociedad Española de Trasplante Hepático (ILTS-SETH) consensus conference guidelines state that LT recipients with risk factors such as alcohol consumption, smoking, and human papillomavirus (HPV) infection should undergo annual HNC surveillance, including oral and ear-nose-throat exams (1). Renaud et al. compared two cohorts of patients who underwent LT for alcoholic liver disease (ALD) and found that intensive surveillance did not favorably impact the stage of HNC at diagnosis or survival (7).

This study aimed to evaluate the impact of HNC surveillance on tumor stage at diagnosis and patient survival.

## Patients and methods

We retrospectively analyzed patients who underwent LT at a single center between January 2000 and January 2023. All included patients had a smoking history above 20 pack-years, and either currently smoked or quit smoking less than 10 years before. These patients underwent annual post-LT HNC surveillance by an ear-nose-throat specialist, including oral and neck examination and fiberoptic laryngoscopy. They were followed until November 2023.

Survival free of HNC rates were calculated using the Kaplan-Meier method; for this purpose, living or dead patients who did not have HNC were censored at their last follow-up visit. The potential influence of various factors on this risk were analyzed using the log-rank product-limit method for categorical variables and Cox regression for continuous variables. The factors studied were age, gender, ALD before LT, hepatocellular carcinoma before LT, Model for End-stage Liver Disease (MELD) score at transplantation, cumulative smoking before LT (above or below 40 pack-years) and whether the patient was currently smoking at the time of LT.

The following data were recorded for each patient diagnosed with HNC: location of the cancer, TNM stage, treatment received, and whether recurrence occurrence during the follow-up period. The survival rates after diagnosis of HNC were calculated using the Kaplan-Meier method. We also recorded whether the surveillance program was adequately followed for each patient diagnosed with HNC. The investigators arbitrarily defined that the surveillance program was adequately followed if the interval between the last surveillance visit and the date of HNC diagnosis was below two years.

Immunosuppressive therapy suffered several changes in the period of the study. Between 2000 and 2004, patients received triple therapy based on the combination of cyclosporine or tacrolimus, azathioprine and steroids. From 2004 to 2011, most patients received tacrolimus, mycophenolate mofetil (MMF) and steroids. From 2011 to date, most patients received tacrolimus and MMF, without steroids. Target levels of tacrolimus were 10 ng/mL in the first three months after LT, 6-9 ng/mL in the months 4 to 6, 5 ng/mL at the end of the first year and 3-5 ng/mL thereafter. In patients with chronic kidney disease, calcineurin inhibitors were reduced or even withdrawn in the long term. Sirolimus or everolimus were used in patients who had been transplanted for hepatocellular carcinoma and had microscopic vascular invasion, and in patients who had pos-transplant malignancy, unless they had significant proteinuria. Patients who required antineoplastic chemotherapy received tacrolimus monotherapy, to avoid the potential additive myelotoxicity of chemotherapy and immunosuppressive drugs.

Continuous variables were described as median (interquartile range, IQR) and categorical variables as number (%). The hazard ratio and 95% confidence interval were obtained using univariate Cox regression. The statistical software used for all analyses was SPSS for Windows 20.0 (IBM Corp., Armonk, NY).

The study protocol was approved by the local institutional review board with the code 2023.28-TFG. Because the study was retrospective, the requirement for informed consent was waived. The clinical data were accessed through anonymized access to the electronic health records. All research was conducted in accordance with the Declarations of Helsinki and Istanbul.

## Results

During the study period, 457 patients underwent their first LT at our institution. 119 (26%) of them had a smoking history of more than 20 pack-years and were the study population. None of them had received HPV vaccination. **Table 1** shows their general features.

**Table 1:** Characteristics of the 119 liver transplant patients with a smoking history above 20 pack-years (2000-2023).

Age (years)	58 (53-63)
Gender Male Female	109 (91.6%) 10 (8.4%)
Alcoholic liver disease	75 (63.0%)
Hepatocellular carcinoma	52 (43.7%)
MELD* score	15 (11-18)
Cumulative smoking 20-40 pack-years Above 40 pack-years	59 (49.6%) 60 (50.4%)
Actively smoking	50 (42.0%)
Follow-up (months)	93 (51-139)

*MELD, Model for End-stage Liver Disease.

**Figure 1:**
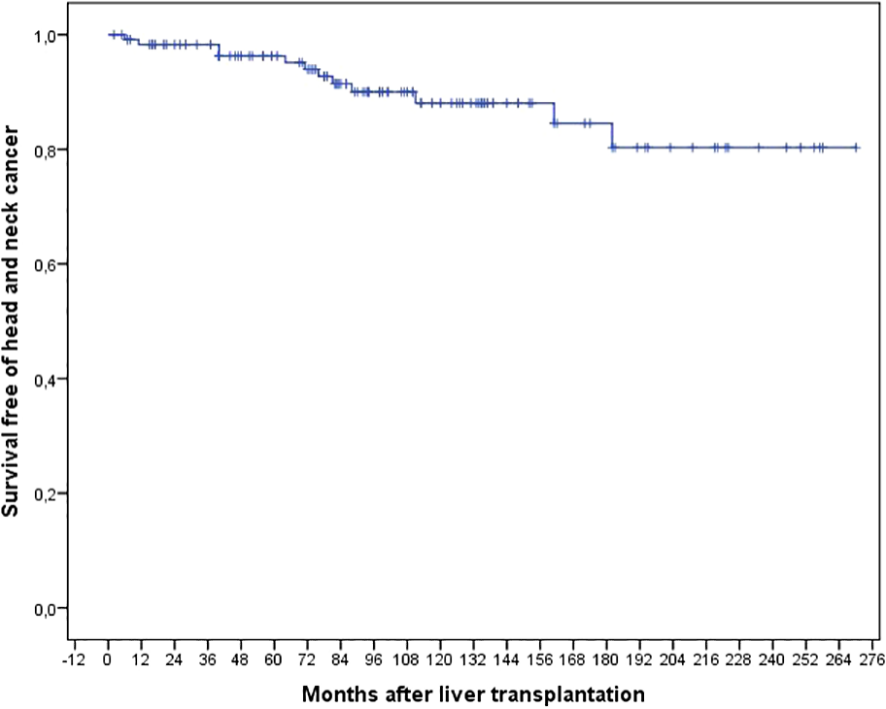
Actuarial risk of survival free of head and neck cancer after liver transplantation in 119 patients with a smoking history above 20 pack-years (2000-2023).

Twelve patients (10.1%) were diagnosed with HNC. The actuarial rates of survival free of HNC at 1, 3, 5, and 10 years were 98.8%, 98.8%, 96.7% and 87.1%, respectively, as shown in **Figure 1**.

**Figure 2:**
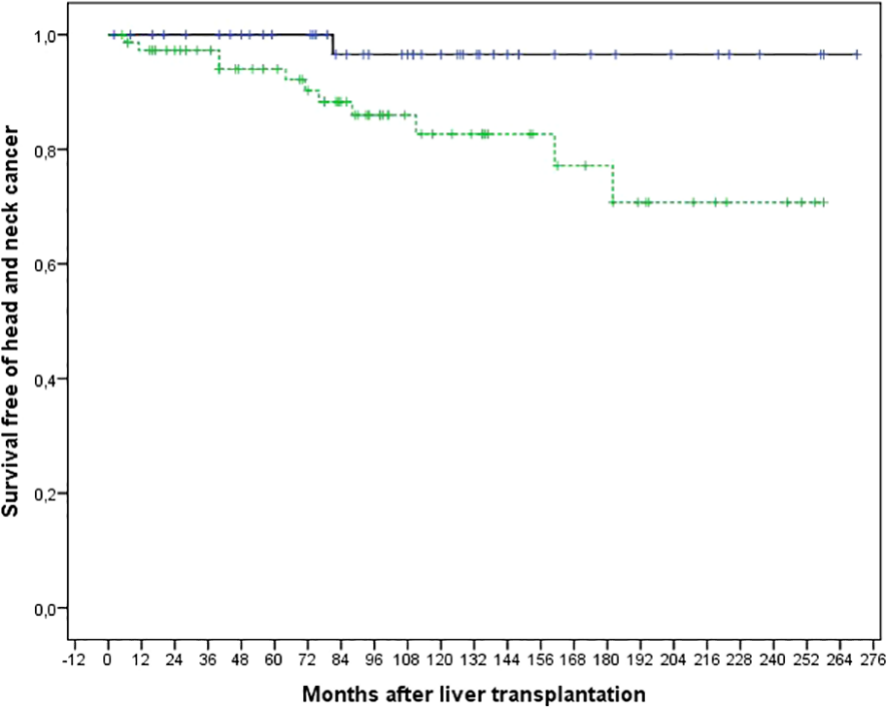
Comparison of the actuarial risk of survival free of head and neck cancer after liver transplantation in 75 patients transplanted for alcoholic liver disease (dotted line) and 44 patients transplanted for liver disease of another origin (continuous line) (p=0.026).

The 5- and 10-year rates of survival free of HNC for patients who had been transplanted for ALD were 94.0% and 82.7%, respectively. In comparison, the rates for patients who had undergone LT for other causes were 100% and 96.6%, respectively (p = 0.026). as shown in **Figure 2**. The only factor associated with a lower risk of survival free of HNC was to have been transplanted for ALD, as indicated in **Table 2**.

**Table 2:** Univariate analysis of factors associated to survival free of head and neck cancer in 119 liver transplant patients with a smoking history above 20 pack-years (2000-2023).

Risk factor	HR (95% CI)*	p
Male gender	1.37 (0.18-11.11)	0.76
Age (years)	0.97 (0.90-1.05)	0.49
Alcoholic liver disease	0.14 (0.02-1.06)	0.06
Hepatocellular carcinoma	0.65 (0.21-2.00)	0.45
MELD** score	0.93 (0.83-1.04)	0.24
Active smoking	0.76 (0.25-2.38)	0.64
Smoking history above 40 pack-years	1.22 (0.38-3.85)	0.74

*Hazard ratio (95% confidence interval).

**MELD, Model for End-stage Liver Disease.

All of the cases of HNC were identified as squamous-cell carcinomas. HPV was not detected in any of them. **Table 3** outlines the location, stage, initial therapy, recurrence, status, and details about the patient’s most recent surveillance visit before the cancer diagnosis.

**Table 3:** Detailed information on 12 head and neck cancers diagnosed in 119 patients with a smoking history above 20 pack-years (2000-2023).

Location	Time after LT (months)	TNM Stage***	Treatment	Recurrence	Current status	Survival (months)	Previous surveillance*
Tongue	182	II	Surgery	No	Dead	63	12 months
Oral cavity	71	Unknown	Surgery	No	Dead	23	12 months
Larynx	11	0	Surgery	No	Alive	223	
Oral cavity	161	IVa	Surgery + CTX + RX**	Yes	Alive	60	60 months
Larynx	76	IVa	Surgery + RX**	No	Dead	22	48 months
Larynx	81	I	Surgery	No	Alive	66	12 months
Larynx	88	II	Surgery + RX**	No	Alive	38	24 months
Hypopharynx	40	IVc	CTX**	No	Dead	32	12 months
Tongue	7	IVa	Surgery + RX**	No	Alive	122	
Larynx	111	I	Surgery + Rx**	Yes	Alive	62	14 months
Larynx	40	IVa	Surgery + CTX + RX**	No	Dead	17	7 months
Larynx	64	0	Surgery	Yes	Alive	65	12 months

*Time between last surveillance visit and the diagnosis of head and neck cancer (two patients were diagnosed at their first surveillance visit).

**CTX, Chemotherapy; RX, Radiotherapy.

***TNM stage of the first head and neck cancer diagnosis (three patients had recurrences).

Of the 12 cases, 4 were located in the oral cavity (including the tongue), one in the hypopharynx and 7 in the larynx. The staging information was unavailable for one patient, who was treated at another medical center. Among the remaining 11, 6 were diagnosed at an early or intermediate stage (0, I, or II), while 5 were diagnosed at an advanced stage (IV). The only 2 patients who did not follow the surveillance program were diagnosed at an advanced stage. Two other patients were diagnosed at an advanced stage despite undergoing regular surveillance. Two patients were diagnosed during their first surveillance visit within the first post-LT year (one at an early stage and one at an advanced stage).

Three patients experienced recurrent disease. One of them, who had stage IV oral cavity malignancy at diagnosis, experienced stage II recurrence in the oral cavity 50 months after the initial diagnosis. The second patient, who had stage I larynx carcinoma, experienced three *in situ* recurrences (9, 36, and 45 months after the initial diagnosis) and a stage II recurrence at 59 months. The third patient, who had *in situ* laryngeal carcinoma, experienced a stage I recurrence 25 months later and a stage IV recurrence at 57 months. It’s worth noting that all of the patients with recurrent disease continued to smoke after their HNC diagnosis.

The first treatment approach was surgery in most patients. Only one patient with stage IVc HNC received only antineoplastic chemotherapy. Surgical therapy included lymphadenectomy in six cases. Five of them also received postoperative external radiotherapy as part of the treatment (two of them received it for the treatment of recurrences, not of primary tumor) and two patients received postoperative radiotherapy and chemotherapy.

**Figure 3:**
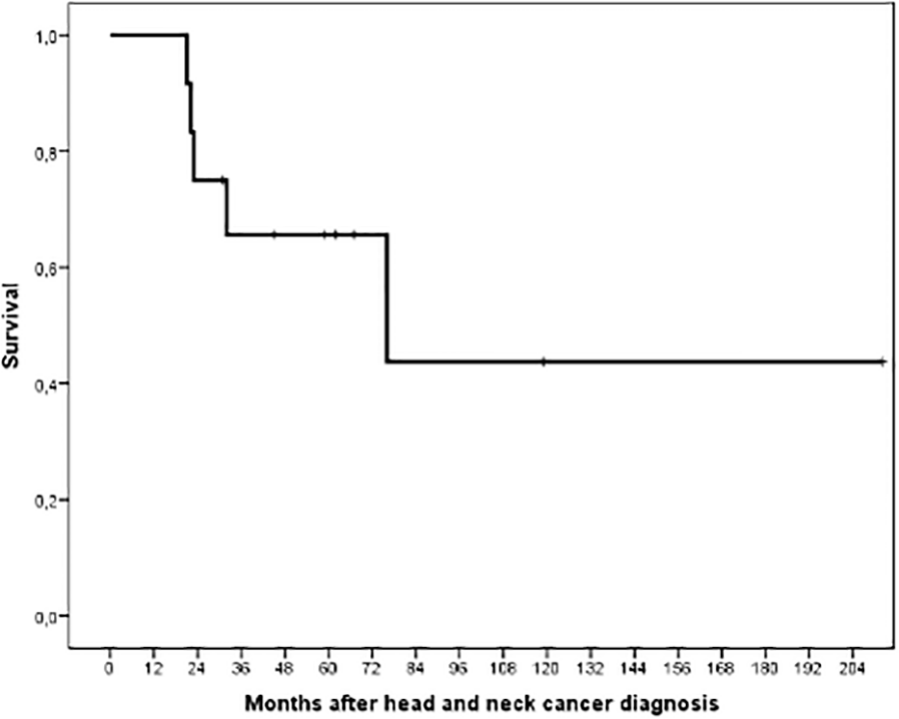
Actuarial survival following the diagnosis of head and neck cancer in 12 patients.

At the last follow-up, five patients had passed away, while seven remained alive. Among the patients who passed away, three perished secondary to HNC progression, while two passed away due to lung and urologic cancers. According to the report, the five-year survival rate after the initial diagnosis was 65.6%. The actuarial survival after the first diagnosis of HNC is shown in **Figure 3**.

## Discussion

The most remarkable result of this manuscript is the finding that surveillance of HNC in LT recipients with a cumulative smoking history above 20 pack-years allows early diagnosis, potentially increasing the chance of successful treatment. The only two patients diagnosed with HNC who had not followed the surveillance program, had advanced stage malignances, highlighting the importance of adhering to the ILTS-SETH guidelines, that recommend annual surveillance for high-risk HNC patients (1). Encouraging results have also been found in the surveillance of lung cancer (7, 9).

Unfortunately, our surveillance program has two important drawbacks. First, two patients were diagnosed at an advanced stage despite adequate surveillance. Secondly, two patients were diagnosed in the first post-LT year (one at an advanced stage). It seems likely that this patient, whose HNC was diagnosed 6 months after LT, could have been diagnosed at an earlier stage if pre-LT screening had been performed. Some studies have shown that an ear-nose-throat screening in LT candidates may allow the diagnosis of HNC in a small but significant proportion of patients, mainly if they had ALD and/or were smokers (10–12). Accordingly, our current protocol of malignancy screening before LT includes an ear-nose-throat evaluation in patients with a greater than 20 pack-years smoking history. In the same way, the 2016 European Association for the Study of the Liver guidelines recommend searching for HNC in LT candidates with alcohol and smoking addiction (13).

Another important finding of our study is the critical influence of alcohol consumption on the risk of HNC. Heavy drinking is a well-known risk factor for the development of HNC in the general population (14). This finding may help reinforce surveillance in patients with a higher risk. Another potential approach for patients with a high risk of malignancy is to avoid tacrolimus overexposure because of its potential influence on the risk of *de novo* malignancy (15, 16).

On the contrary, an unexpected finding of our study was the absence of a reduction in the risk of HNC after smoking withdrawal. A previous study found that the risk of smoking-related malignancies was reduced in LT recipients who quit smoking (17). Similarly, the risks of lung and HNC are reduced after smoking withdrawal in the general population (18, 19). In our study, the classification of a patient as a current or past smoker was done at the time of LT. So, patients who relapsed smoking or those who quit smoking after LT may have been misclassified. On the other side, it is interesting to underline that the three patients with cancer recurrence kept smoking, thus suggesting the importance of the programs oriented to smoking cessation in LT recipients by the recommendations in non-transplanted patients after the diagnosis of cancer (20).

Our surveillance protocol has some drawbacks, but it is encouraging that a relevant proportion of the patients were diagnosed at a potentially curable stage, and the actuarial survival rate after the diagnosis of HNC is acceptable. Both compare favorably with most of the series of HNC in LT recipients published to date (2, 6–8). However, further studies are necessary to determine if this annual surveillance visit strategy is valuable and cost-effective. In the general population, oral cancer screening by visual inspection is cost-effective in high-risk populations (21), but the results of HNC screening remain controversial (22).

The study has some limitations that warrant consideration. Firstly, it is retrospective, and our series is limited in size. However, it is worth mentioning that the cohort of patients has been closely followed, and the ear-nose-throat specialist who screened the patients was the same in most cases.

To conclude, head and neck cancer is a frequent complication after liver transplantation in patients who smoke, especially in those who have been transplanted for alcoholic liver disease. Annual screening for head and neck cancer can lead to early diagnosis and improve survival rates following a cancer diagnosis.

## Data Availability

The raw data supporting the conclusions of this article will be made available by the authors, without undue reservation.

## Author’s note

CIBERehd is funded by the Instituto de Salud Carlos III (Madrid, Spain).
